# Local abaloparatide administration promotes in situ alveolar bone augmentation via FAK-mediated periosteal osteogenesis

**DOI:** 10.1038/s41368-025-00392-6

**Published:** 2025-09-02

**Authors:** Ruyi Wang, Yuan Li, Bowen Tan, Shijia Li, Yanting Wu, Yao Chen, Yuran Qian, Haochen Wang, Bo Li, Zhihe Zhao, Quan Yuan, Yu Li

**Affiliations:** 1 State Key Laboratory of Oral Diseases & National Center for Stomatology & National Clinical Research Center for Oral Diseases & Department of Orthodontics, West China Hospital of Stomatology, Sichuan University, Chengdu, China; 2 State Key Laboratory of Oral Diseases & National Center for Stomatology & National Clinical Research Center for Oral Diseases & Department of Oral Implantology, West China Hospital of Stomatology, Sichuan University, Chengdu, China

**Keywords:** Stem-cell differentiation, Bone remodelling, Cell proliferation

## Abstract

Insufficient alveolar bone thickness increases the risk of periodontal dehiscence and fenestration, especially in orthodontic tooth movement. Abaloparatide (ABL), a synthetic analog of human PTHrP (1–34) and a clinical medication for treating osteoporosis, has recently demonstrated its potential in enhancing craniofacial bone formation. Herein, we show that intraoral submucosal injection of ABL, when combined with mechanical force, promotes in situ alveolar bone thickening. The newly formed bone is primarily located outside the original compact bone, implying its origin from the periosteum. RNA sequencing of the alveolar bone tissue revealed that the focal adhesion (FA) pathway potentially mediates this bioprocess. Local injection of ABL alone enhances cell proliferation, collagen synthesis, and phosphorylation of focal adhesion kinase (FAK) in the alveolar periosteum; when ABL is combined with mechanical force, the FAK expression is upregulated, in line with the accomplishment of the ossification. In vitro, ABL enhances proliferation, migration, and FAK phosphorylation in periosteal stem cells. Furthermore, the pro-osteogenic effects of ABL on alveolar bone are entirely blocked when FAK activity is inhibited by a specific inhibitor. In summary, abaloparatide combined with mechanical force promotes alveolar bone formation via FAK-mediated periosteal osteogenesis. Thus, we have introduced a promising therapeutic approach for drug-induced in situ alveolar bone augmentation, which may prevent or repair the detrimental periodontal dehiscence, holding significant potential in dentistry.

## Introduction

Bone formation initiates in the process of intramembranous or endochondral ossification. Craniofacial bones, such as the mandibles and maxillae, principally originate from the former, while the limb bones originate from the latter.^1^ Despite their varied formation processes, bones undergo remodeling and modeling in response to mechanical force. Typically, appropriate mechanical loading promotes bone gain, while its absence or reduction triggers bone loss.^1^ The alveolar bone, an integral component of the jawbone, is renowned for its exceptional remodeling capacity in response to mastication force, as well as its modeling potential under orthodontic force.^2,3^ Notably, different from bone at other anatomical sites, the alveolar bone is devoid of muscular tissue and is instead covered by a mere layer of gingiva, a characteristic that renders it highly conducive for local drug delivery.

Parathyroid hormone-related protein (PTHrP) is a locally acting auto/paracrine ligand, sharing the type 1 PTH/PTHrP receptor (PTH1R) with parathyroid hormone (PTH). PTHrP plays essential roles both in limb bones and alveolar bone.^4^ In developing limb bones, PTHrP-positive chondrocytes in the resting zone continue to form columnar chondrocytes, thereby expanding precursor cells for endochondral ossification.^5^ PTHrP in the dental follicle is required for tooth eruption, through activating alveolar bone resorption and/or formation.^6^ PTHrP is also upregulated in the periodontal ligament in response to orthodontic force.^7^

Abaloparatide (ABL), a synthetic analog of human PTHrP (1–34), has garnered approval, following teriparatide (PTH1-34), as a clinical medication for treating osteoporosis. PTH, PTHrP, and ABL exhibit a similarly high binding affinity for the RG conformation of PTH1R, whereas the affinity for the R0 conformation differs among them, being higher for PTH, followed by PTHrP and then ABL.^8,9^ Activation of the RG conformation is associated with shorter and more transient signaling responses, beneficial for bone formation, while activation of the R0 conformation leads to longer and more sustained signaling, potentially stimulating bone resorption.^10^ Hence, ABL selectively activates the RG conformation, shifting the balance toward bone formation rather than resorption. Although the underlying mechanisms remain unclear,^8^ ABL has demonstrated osteoanabolic effects in limb bones superior to its predecessor teriparatide in animal and clinical studies.^11–13^

While ABL is intentionally developed for treating osteoporosis, its potential application in the oral region has just burgeoned. In an experimental periodontitis rat model, we found that systemic administration of ABL protects against the alveolar bone loss by enhancing osteogenesis, with the effect superior to teriparatide.^14^ Phosphorus nanoflowers loaded with ABL promote alveolar bone regeneration in the tooth extraction sockets.^15^ Both daily subcutaneous injection of ABL^16^ and nanocoating embedded with ABL enhance osseointegration of dental implants.^17^ We also found that systemic administration of ABL enhances mandibular growth in adolescent rats, with better effects than teriparatide.^18^ Additionally, in the group wearing an inclined bite splint, which imposes mechanical force on the mandibular incisors, systemic administration of ABL significantly increases the labial alveolar bone thickness.

Alveolar bone volume is crucial for periodontal health. Insufficient alveolar bone thickness increases the risks of periodontal dehiscence and fenestration.^19^ Specifically, alveolar bone dehiscence is intimately linked to gingival recession, an intractable periodontal defect.^20^ Moreover, orthodontic tooth movement may reduce the surrounding alveolar bone.^21^ When the native alveolar bone thickness is inadequate while labial or buccal tooth movement is needed, the only available clinical management to alleviate dehiscence appears to be bone grafting surgeries.^22^ Aside from their cost, discomfort, and potential side effects, the increase in alveolar bone thickness may be limited following such surgeries.^23^

The preliminary finding in our previous study^18^ has illuminated the potential of ABL to augment alveolar bone, while two critical questions must be addressed as a priority. First, since systemic administration of ABL is apparently unsuitable for this localized objective, will local injection of the drug be effective? Second, as the incisor root of rats is quite different from that of humans, can the anticipated outcome be translated similarly to the buccal alveolar bone of the rat molars? Therefore, this study investigated the effects of local ABL administration on both the labial and buccal alveolar bone in rat models subjected to orthodontic tooth movement.

Herein, we demonstrated that local submucosal ABL injection, when combined with mechanical force, effectively promotes labial and buccal alveolar bone augmentation through periosteal osteogenesis. Mechanistically, ABL alone enhances proliferation and FAK phosphorylation in the periosteal cells, while ABL combined with mechanical force upregulates the FAK expression, in line with the accomplishment of ossification. Furthermore, FAK inhibition abolishes the ABL-induced periosteal osteogenesis in the alveolar bone. Thus, we propose the drug-induced in situ alveolar bone augmentation as a potential approach to prevent or repair the detrimental periodontal dehiscence, offering a possible complement to the current practice of bone grafting. Meanwhile, the revealed pivotal role of FAK in mediating the ABL-induced and mechano-dependent osteogenesis in alveolar bone has expanded understanding of the osteoanabolic synergy elicited by integrating the PTH1R and mechanical signals.

## Results

### Local injection of ABL augments the labial alveolar bone only when combined with orthodontic force

We established a labial movement (LM) model using an inclined bite splint previously reported^18^ to move the rat mandibular incisors labially (Fig. S1a). Local submucosal injection of ABL at the labial vestibule was given in combination with/without the LM force. The dosage was determined as 20 μg/kg for the local injection of ABL, according to the results in the pilot study (Figs. S2, S3). After a 2-week intervention, the labial alveolar bone was assessed using micro-CT at the crest and mid-ridge levels, respectively (Fig. S1b).

**Fig. 1:**
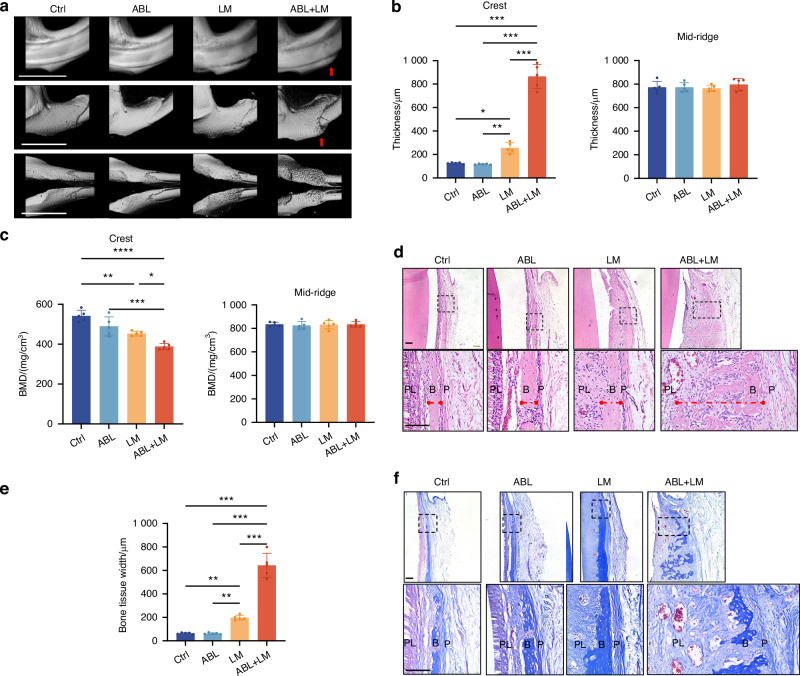
Local injection of ABL augments the mandibular labial alveolar bone only when combined with orthodontic force. **a** Micro-CT images. Red arrow: the thickening of the labial alveolar bone in the ABL + LM group. Scale bar: 5 mm. Alveolar bone thickness (**b**) and BMD (**c**) at the crest and mid-ridge levels. **d** HE staining. Red dotted line: the bone tissue width. Scale bar: 100 μm. **e** Quantification of the bone tissue width. **f** Masson staining. Scale bar: 100 μm. PL periodontal ligament, B alveolar bone, P periosteum. *n* = 5 per group. *P < 0.05, **P < 0.01, ***P < 0.001

Micro-CT analysis (Fig. 1a–c) showed that the ABL + LM group had significantly increased labial alveolar bone thickness at the crest level compared to other groups, along with significantly lower bone mineral density (BMD). No significant differences were observed among the groups at the mid-ridge level (Fig. 1b, c). Notably, the alveolar bone thickness in the ABL + LM group is 865.40 μm ± 103.28 μm, about 6.9 times that of the control (Ctrl) group and 3.4 times that of the LM group (Fig. 1b).

HE staining (Fig. 1d, e) showed that the ABL + LM group had dramatically increased bone tissue width compared to other groups, with abundant periosteal cells and woven bone tissue. The ABL group appeared to have increased cell number in the periosteal cambial layer compared to the Ctrl; however, no significant increase in the bone tissue width was observed. Masson staining (Fig. 1f) showed that in the ABL + LM group, collagen fibers increased in the periosteum and extended into the bone tissue. Immunohistochemical (IHC) staining (Fig. S4a, b) showed that ABL, whether combined with the LM force or not, upregulated the expression of COL1A1 and Ki-67 in the periosteum, suggesting that it alone can promote the periosteal cell proliferation and collagen synthesis. Nevertheless, OCN was upregulated only in the ABL + LM group (Fig. S4c), suggesting that the mechanical force is indispensable for the ABL-induced ossification in the alveolar bone.

Thus, local submucosal injection of ABL combined with the orthodontic force pronouncedly increases the labial alveolar bone thickness through in situ osteogenesis; in contrast, ABL alone only promotes periosteal cell proliferation and collagen synthesis, without the accomplishment of ossification.

### Local injection of ABL differentially augments the buccal alveolar bone with or without orthodontic force

Considering the disparity between the incisor root of the rat and that of humans, we wonder whether the desired effect of ABL is unique at the rat labial area or not. Therefore, we next employed a buccal movement (BM) model using a maxillary expansion device to apply buccal force on the maxillary molars (Fig. S1c).

**Fig. 2:**
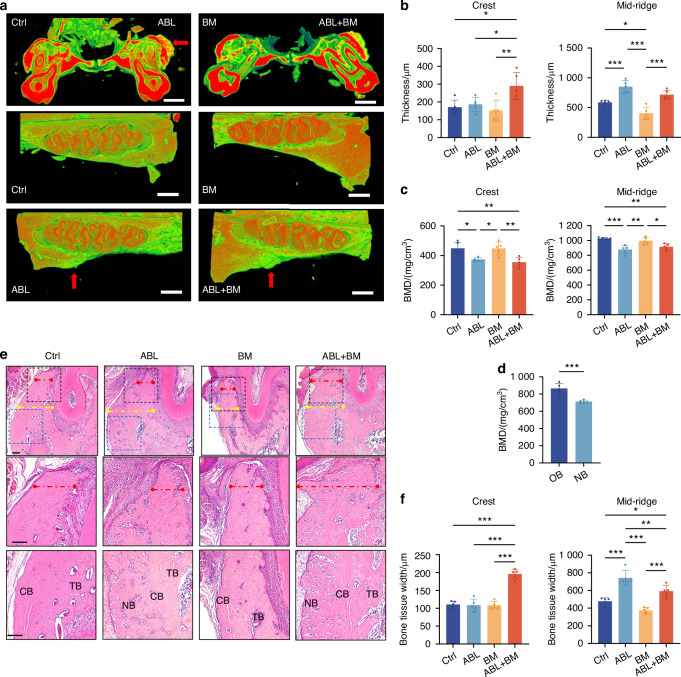
Local injection of ABL differentially augments the buccal alveolar bone with or without orthodontic force. **a** Micro-CT images. Red arrow: the newly formed bone of lower density. Scale bar: 1 mm. Alveolar bone thickness (**b**) and BMD (**c**) at the crest and mid-ridge levels. **d** BMD of the newly formed bone (NB) and the original bone (OB) in the ABL group. **e** HE staining. Bone tissue width at crest (red dotted line) and mid-ridge (blue dotted line) levels was quantified in (**f**). Scale bar: 100 μm. TB trabecular bone, CB compact bone, NB newly formed bone. n = 5 per group. *P < 0.05, **P < 0.01, ***P < 0.001

After a 2-week intervention, the buccal alveolar bone was assessed using micro-CT at the crest and mid-ridge levels, respectively (Fig. S1d). At the crest level, the ABL + BM group showed significantly increased thickness compared to other groups (Fig. 2a, b), consistent with the results in the mandibular LM model. Unexpectedly, at the mid-ridge level, the ABL group had significantly increased thickness (850.00 μm ± 103.58 μm) compared to the Ctrl (585.00 μm ± 37.94 μm) (Fig. 2b), along with decreased BMD (Fig. 2c). The newly formed bone of lower density (Fig. 2d) was observed on the outside of the original bone with a clear boundary between them (Fig. 2a). These indicate that ABL alone promotes the buccal alveolar bone thickening at the mid-ridge level, and the newly formed bone may well originate from the periosteum.

HE staining (Fig. 2e, f) revealed increased bone tissue width, coincident with the micro-CT findings. The newly formed bone in the ABL and ABL + BM groups, taking the morphology of trabecular bone, appeared on the outer surface of the compact bone, further implying its origin from the periosteum. IHC staining (Fig. S5a, b) showed that the ABL and ABL + BM groups had significantly upregulated expression of COL1A1 and Ki-67 within the periosteum, consistent with the results in the labial model above. The difference was that here, ABL alone, independent of the orthodontic force, also significantly upregulated OCN (Fig. S5c), coincident with the buccal alveolar bone augmentation.

The differential effects of ABL alone on the mandibular labial and maxillary buccal alveolar bone are thought-provoking. Due to mastication, the buccal alveolar bone undergoes a much higher level of physiological mechanical stimulation compared to the labial counterpart. Therefore, local injection of ABL at the buccal vestibule may well take advantage of the masticatory force to fulfill its pro-osteogenic task. In contrast, ABL alone has no substantial effects on the labial alveolar bone, where the constitutive mechanical strain is much lower, making the orthodontic force indispensable to accomplish the ossification. The different locations of pronounced buccal bone augmentation, namely at the mid-ridge vs. crest level, in the ABL and ABL + BM groups, respectively, might reflect the different focuses of the occlusal vs. orthodontic force. Furthermore, a supplementary experiment of unilateral mandibular molar extraction revealed that ABL injection alone could no longer enhance maxillary buccal alveolar bone thickening on the extraction side, probably due to the loss of occlusal force (Fig. S6).

Taken together, local injection of ABL promotes in situ osteogenesis at the buccal alveolar bone independent of orthodontic force, with the newly formed bone located outside the compact bone, once again suggesting its periosteal origin.

### RNA-seq revealed that FAK potentially mediates the alveolar bone osteogenesis induced by ABL combined with force

The findings above highlight the mandibular labial alveolar bone as a native region of low-level constitutive mechanical stimulation, making it an appropriate site to elucidate the synergy of ABL and mechanical force. Hence, we analyzed the mandibular labial alveolar bone tissues in the Ctrl, LM, ABL, and ABL + LM groups via mRNA sequencing (RNA-seq).

Heatmap exhibited differentially expressed genes (DEGs, Fig. S7a). Gene Ontology (GO) analysis of DEGs revealed significant enrichment of biologically relevant GO terms in the ABL + LM group, including positive regulation of cell migration (GO:0030335), collagen fibril organization (GO:0030199), skeletal system development (GO:0001501), and bone mineralization (GO:0030282) (Fig. S7b).

**Fig. 3:**
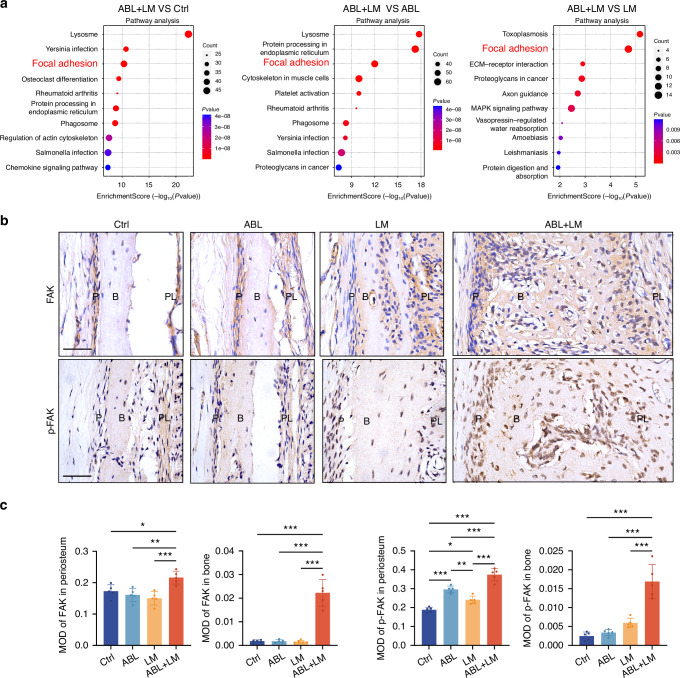
The FAK expression is upregulated by ABL only when it is combined with sufficient mechanical force. **a** KEGG pathway enrichment analysis. Focal adhesion is among the top five pathways enriched in the ABL + LM group compared to other groups. **b** IHC staining of FAK and p-FAK in mandibular labial alveolar bone. Scale bar: 100 μm. **c** Quantification of the expression of FAK and p-FAK in periosteum and bone tissue. PL periodontal ligament, B alveolar bone, P periosteum. n = 5 per group. *P < 0.05, **P < 0.01, ***P < 0.001

Notably, the Kyoto Encyclopedia of Genes and Genomes (KEGG) pathway enrichment analysis (Fig. 3a) revealed that focal adhesion (FA) is among the top five upregulated pathways enriched in the ABL + LM group compared to other groups, indicating its potential roles in integrating the stimuli from ABL and mechanical force. Specifically, focal adhesion kinase (FAK), a key signaling protein of the FA assembly, is upregulated in the ABL + LM group. Coincidentally, in the RNA-seq analysis for the BM model, FA is also within the top five significant pathways in the ABL groups compared to the control (Fig. S8a).

Thus, the potential roles of the FA pathway and its key protein FAK are highlighted in the alveolar periosteal osteogenesis inspired by ABL.

### ABL upregulates the FAK expression in the periosteum only when combined with mechanical force

Enlightened by the findings above, we then performed IHC staining for FAK and phosphorylated FAK (p-FAK). In the labial model, ABL or force alone significantly increased p-FAK levels in the periosteum, while only when they were combined did FAK expression become significantly upregulated in both the periosteum and the bone (Fig. 3b, c). In contrast, both ABL + BM and ABL alone significantly upregulated the FAK expression in the buccal model (Fig. S8b, c).

Taken together, ABL combined with sufficient mechanical force upregulates the FAK expression in the periosteum and bone, in line with the accomplishment of the in situ ossification.

### ABL enhances proliferation, migration, and FAK phosphorylation in PSCs

Since the ABL-induced newly formed bone may well originate from periosteum, we proceeded to culture rat periosteal stem cells (PSCs) (Fig. S9a). Flow cytometry analysis demonstrated that these cells expressed cathepsin K (CTSK) and typical mesenchymal stem cell markers (CD90, CD29, and CD44), while lacking the hematopoietic marker CD45 (Fig. S9b). Multidirectional differentiation potentials of the PSCs were verified with alkaline phosphatase (ALP) and alizarin red S (ARS) staining, oil red O staining, and alcian blue staining, respectively (Fig. S9c).

**Fig. 4:**
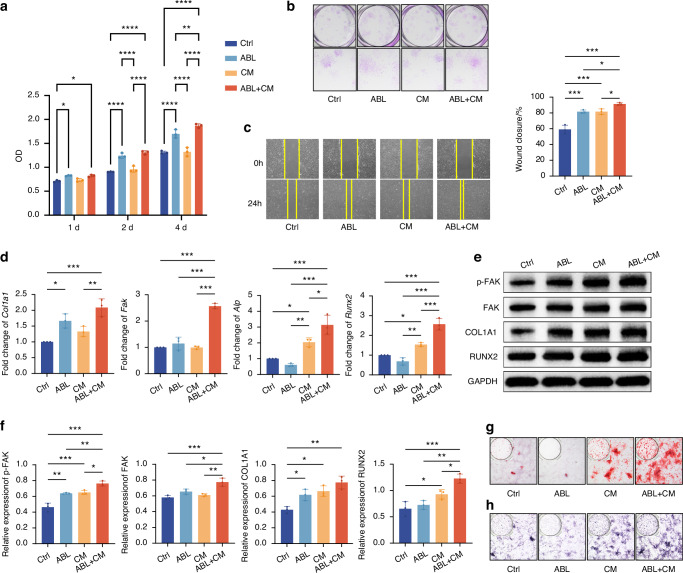
ABL enhances proliferation, migration, collagen synthesis, and FAK phosphorylation in PSCs. **a** CCK-8 assay at 1, 2, and 4 d. **b** CFU assay at 4 d. **c** Wound healing assay at 24 h. **d** Relative expression of mRNA (*Col1a1, Alp, Runx2*, and *Fak*). **e** Protein levels of p-FAK, FAK, COL1A1 and RUNX2, with quantification in (**f**). **g** ARS staining. **h** ALP staining. n = 3 per group. *P < 0.05, **P < 0.01, ***P < 0.001

Cell-counting kit-8 (CCK-8), colony-forming unit (CFU) assay, and wound healing assay demonstrated that ABL promoted the proliferation and migration of PSCs (Fig. 4a–c). Furthermore, ABL significantly upregulated the mRNA expression of *Col1a1* (Fig. 4d), as well as the protein expression of COL1A1 and p-FAK (Fig. 4e, f) in the PSCs.

Nevertheless, ABL suppressed mineralization in the PSCs (Fig. 4g, h). Considering the adjacency of the periosteum with the compact bone in vivo, we then employed the conditioned media (CM) from osteocyte-like MLO-Y4 cells. The CM alone promoted mineralization in the PSCs, with the effect being even stronger when combined with ABL (Fig. 4g, h), in line with the expression of *Alp* and RUNX2 (Fig. 4d–f). Notably, the FAK expression was also significantly upregulated in the ABL + CM group compared to the ABL group (Fig. 4d–f). These suggest that ABL may need the paracrine signals from the osteocytes to accomplish the alveolar bone periosteal osteogenesis.

Altogether, ABL alone enhances PSC accumulation and collagen synthesis in vitro, along with FAK phosphorylation; ABL combined with the osteocytic conditioned media promotes PSC mineralization, along with upregulation of FAK expression.

### The PSC accumulation effects of ABL are nullified by the inhibition of FAK activity

**Fig. 5:**
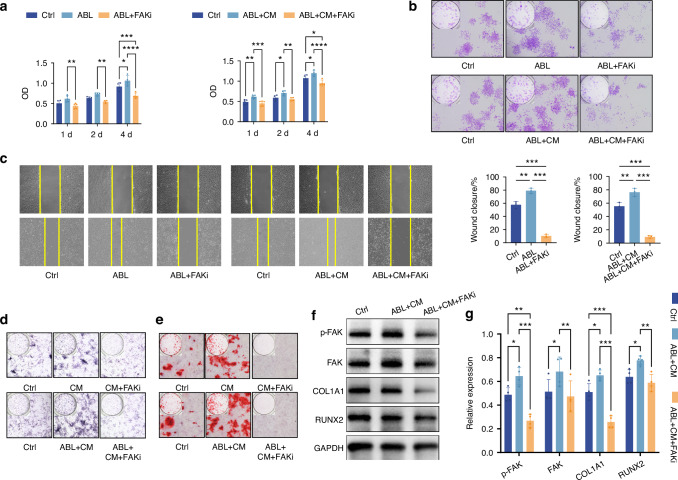
The PSC accumulation effects of ABL are nullified by inhibition of FAK activity. **a** CCK-8 assay at 1, 2, and 4 d. (n = 5 for 1 d; n = 4 for 2 and 4 d) **b** CFU assay at 4 d. **c** Wound healing assay at 24 h. (n = 3) **d** ALP staining. **e** ARS staining. **f** Protein levels of p-FAK, FAK, COL1A1 and RUNX2, with quantification in (**g**). (n = 4). *P < 0.05, **P < 0.01, ***P < 0.001

To investigate the roles of FAK, we applied FAK inhibition (FAKi) with a FAK phosphorylation inhibitor (PF-573228) before treating the PSCs with ABL. FAKi reversed the proliferative (Fig. 5a, b) and pro-migration (Fig. 5c) effects of ABL on the PSCs. Furthermore, the pro-mineralization effect of ABL + CM was also reversed by FAKi (Fig. 5d, e), accompanied by a decrease in the expression of COL1A1 and RUNX2 (Fig. 5f, g).

Overall, the in vitro PSC accumulation effects of ABL are dependent on the activation of FAK.

### The pro-osteogenic effects of ABL are abolished in alveolar bone and partially suppressed in limb bone by inhibition of FAK activity

**Fig. 6:**
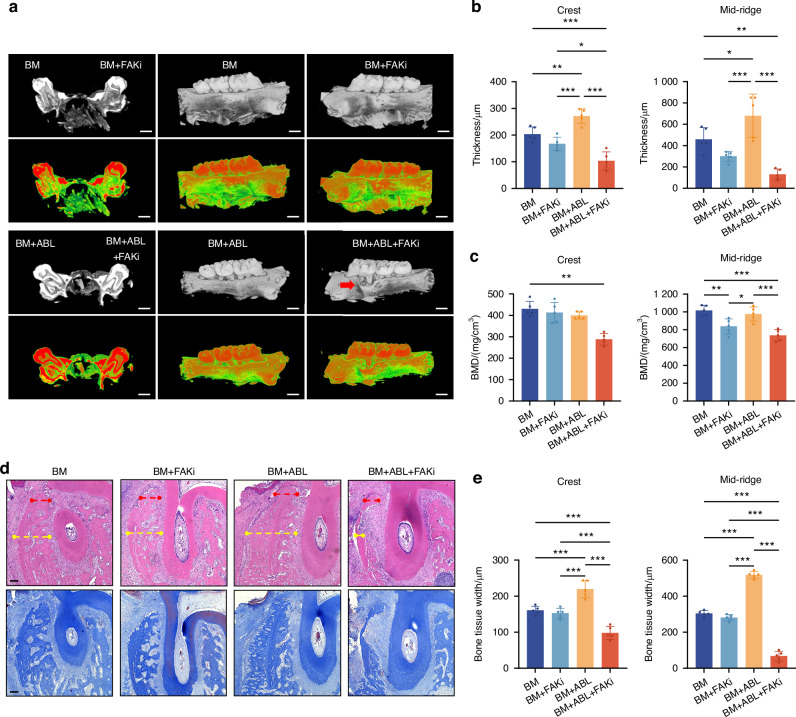
The pro-osteogenic effects of ABL are abolished in alveolar bone by inhibition of FAK activity. **a** Micro-CT images of maxillae in BM models. Red arrow: alveolar bone dehiscence in BM + ABL+FAKi group. Scale bar: 1 mm. Buccal alveolar bone thickness (**b**) and BMD (**c**) at the crest and mid-ridge levels. **d** HE and Masson staining. Bone tissue width at crest (red dotted line) and mid-ridge (yellow dotted line) levels was quantified in (**e**). Scale bar: 100 μm. n = 5 per group. *P < 0.05, **P < 0.01, ***P < 0.001

**Fig. 7:**
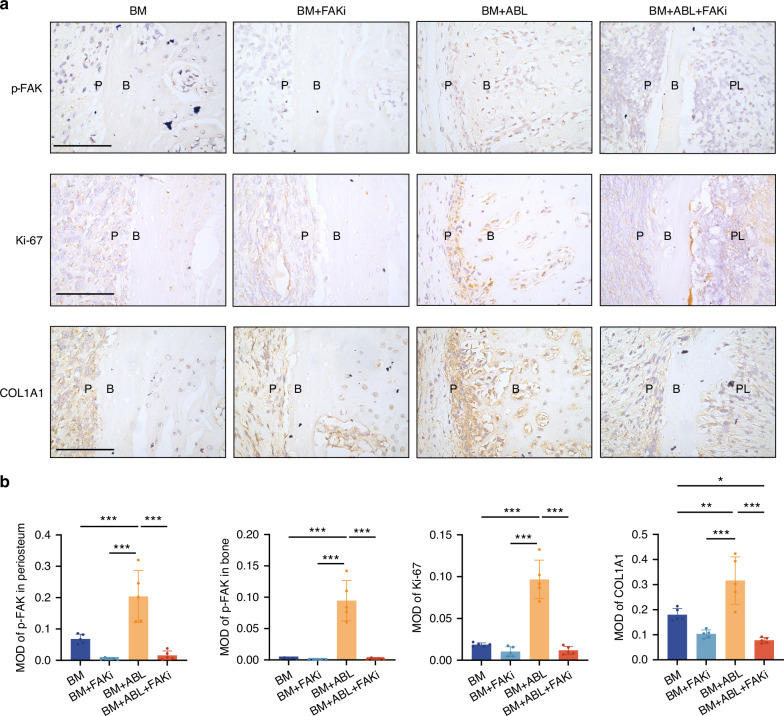
Inhibition of FAK activity affects the anabolic effect of ABL. **a** IHC staining of p-FAK, Ki-67 and COL1A1, with quantification in (**b**). Scale bar: 100 μm. PL periodontal ligament, B alveolar bone, P periosteum. n = 5 per group. *P < 0.05, **P < 0.01, ***P < 0.001

To further verify the role of FAK activity in the ABL-induced periosteal osteogenesis in alveolar bone, we locally administered PF-573228 for FAKi before injection of ABL in the buccal model. The BM + ABL+FAKi group suffered from striking buccal alveolar bone dehiscence (Fig. 6a), with reduced bone thickness compared to other groups and lower BMD compared to the Ctrl (Fig. 6b, c). HE and Masson staining (Fig. 6d, e) revealed a significant reduction in alveolar bone tissue width in the BM + ABL+FAKi group, with no bone formation observed. Additionally, the BM + ABL group had increased tartrate-resistant acid phosphatase (TRAP)-positive osteoclasts compared to the BM group, while this osteoclastogenic effect of ABL was unaffected by FAKi (Fig. S10). IHC staining demonstrated that the ABL-induced FAK phosphorylation and the upregulation of Ki-67 and COL1A1 were also blocked by FAKi (Fig. 7). Collectively, the pro-osteogenic effects of ABL in alveolar bone are mediated by and dependent on the FAK activity.

**Fig. 8:**
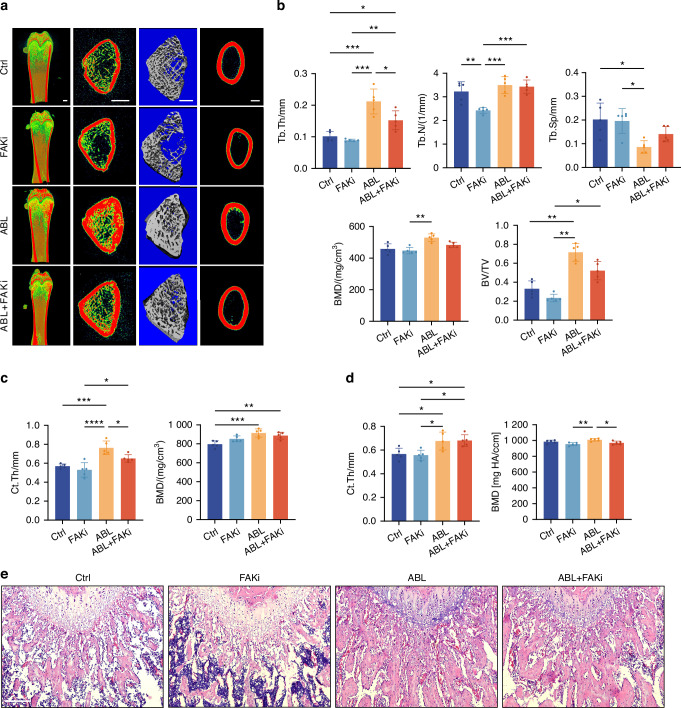
The pro-osteogenic effects of ABL are partially suppressed in limb bone by inhibition of FAK activity. **a** Micro-CT images of the femur. Scale bar: 1 mm. **b** Trabecular analysis of femoral distal metaphysis for trabecular thickness (Tb.Th), trabecular number (Tb.N), trabecular spacing (Tb.Sp), BMD, and bone volume per tissue volume (BV/TV). **c** Cortical analysis of femoral distal metaphysis for cortical thickness (Ct.Th) and BMD. **d** Cortical analysis of femoral diaphysis for Ct.Th and BMD. **e** HE staining of trabecular bone at the femoral distal metaphysis. Scale bar: 200 μm. n = 5 per group. *P < 0.05, **P < 0.01, ***P < 0.001

To investigate whether FAK also plays a role in the ABL-induced osteogenesis in limb bone, we inhibited FAK activity through intraperitoneal injection of PF-573228, followed by subcutaneous administration of ABL. The femoral diaphysis and distal metaphysis were analyzed using micro-CT (Fig. S1e). In the femoral distal metaphysis, ABL significantly increased the cortical bone thickness (Ct.Th) and trabecular thickness (Tb.Th) in the cancellous bone, both partially depressed by FAKi (Fig. 8a–c). In the femoral diaphysis, ABL also significantly increased the cortical bone thickness, which, however, was unaffected by FAKi (Fig. 8d). HE staining showed that ABL increased trabecular number and thickness in the femoral distal metaphysis, which appeared to be partially suppressed by FAKi (Fig. 8e). These indicate that FAK partially regulates the pro-osteogenic effects of ABL in the limb bone.

In summary, the FAK activity plays a pivotal and indispensable role in mediating the pro-osteogenic effects of ABL in alveolar bone, while in limb bone, its role is significant but less crucial.

## Discussion

**Fig. 9:**
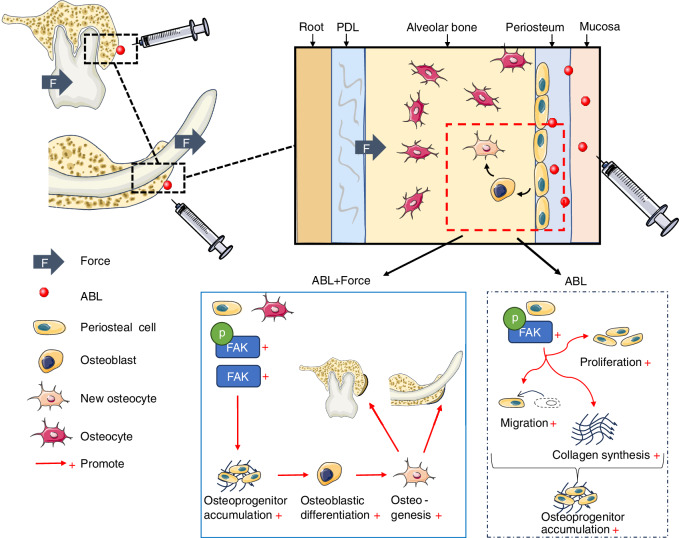
Schematic diagram: Abaloparatide combined with mechanical force promotes in situ alveolar bone augmentation via FAK-mediated periosteal osteogenesis. ABL alone enhances the FAK phosphorylation, which mediates cell proliferation, migration, and collagen synthesis in periosteal cells, resulting in osteoprogenitor accumulation. ABL combined with mechanical force promotes upregulation of FAK in periosteum and bone, which mediates the periosteal osteogenesis that augments the alveolar bone thickness

The osteoanabolic effects of the PTH1R ligand family, including PTH, PTHrP, teriparatide, and abaloparatide, have been extensively investigated in limb bone, with increasing attention paid to maxillofacial bone. Here we show that the local submucosal injection of ABL, when combined with sufficient mechanical stimuli, promotes considerable in situ alveolar bone augmentation through periosteal osteogenesis, which is dependent on the FAK activity (Fig. 9). Thereby, we propose a potential approach for alveolar bone augmentation, which may contribute to preventing and repairing the detrimental periodontal dehiscence, with the possibility of further clinical exploration.

The application of PTH and its derivatives in dentistry has burgeoned. A clinical study published in the *NEJM*^24^ reported that systemic administration of teriparatide can restore alveolar bone defects, indicating its regenerative capacity in the jawbone. Nevertheless, limited advancement has been witnessed in this innovative direction thereafter. Two major reasons might be the concern for the potential side effects and the low benefit-cost ratio of systemic administration of such osteoanabolic drugs to manage localized problems. Hence, intraoral local administration^25^ may be less disadvantageous for the application of these drugs in dentistry.

The local injection of ABL to promote endogenous osteogenesis and thicken the alveolar bone may offer a potential adjunct to existing bone augmentation strategies. The current clinical approach for local osseous augmentation is the bone grafting surgery, using autologous bone, xenograft, allograft, or various artificial biomaterials. However, none of these bone grafts meet all the clinically desirable requirements, including high osteoinductive potentials, low patient morbidity, high volumetric stability, easy market availability, long shelf life, and reasonable production costs.^26^ In contrast, the alveolar periosteal osteogenesis inspired by local ABL injection may offer some practical advantages, including endogenous alveolar bone formation, minimal invasiveness, low technique sensitivity, and relatively low cost, suggesting its potential for clinical translation. Additionally, our pilot study indicated that local submucosal injection of teriparatide (PTH1-34) exhibited similar but less potent effects compared to ABL in enhancing alveolar bone thickness (data not shown).

It has been well documented that the PTH-induced osteogenesis may be intimately associated with mechanical stimuli. In the non-disease mouse model,^27^ the OVX mouse model,^28^ and the diabetic mouse model,^29^ the combination of PTH and mechanical loading elicits more pronounced osteogenesis than either treatment alone, showing a synergistic effect, particularly for periosteal bone formation.^30^ PTH increases cortical thickness in the tibia, which carries a greater mechanical loading, whereas it does not in the less loaded vertebrae.^31^ In bone unloading models constructed by tail suspension^32,33^ or neurectomy,^34^ the osteoanabolic effects of PTH are attenuated. These indicate that mechanical loading is crucial for the osteoanabolic effect of PTH, although the underlying mechanisms remain poorly understood.^8^

Notably, herein we show that mechanical force may also play a pivotal role in the ABL-induced alveolar bone formation. In normal occlusion in humans, the mandibular incisors should have slight or no occlusal contact,^35^ probably bearing only a low level of constitutive mechanical stimuli, manifested with thin alveolar bone in their region. In rats, this characteristic is similar or even more marked, as their mandibular incisors are often positioned posterior to the maxillary incisors with no contact in occlusion. This explains why ABL alone cannot thicken the mandibular labial bone—the absence of sufficient mechanical force. In limb bone, mechanical loading is ubiquitous as required to bear weight and muscle tension, and even unloading by tail suspension or immobilization only partially attenuates the intrinsic mechanical stimuli. Therefore, it is the mandibular labial alveolar bone, a unique area lacking sufficient physiological mechanical stimuli, that unexpectedly exemplifies the critical role of mechanical force in the pro-osteogenic effects of ABL.

Unlike limb bone, the development of the jawbones primarily occurs through intramembranous ossification, driven by the differentiation of periosteal progenitor cells.^36,37^ Human jawbone-derived periosteal stem cells display growth curves similar to those from tibia, with equal tri-lineage differentiation potential.^38^ Furthermore, the neural crest-derived stem cells residing within the cambium layer of the periosteum are renowned for fulfilling in situ osteogenesis to repair jawbone defects.^39^ We have identified that the local ABL-induced alveolar bone formation primarily originates from the periosteum, presenting a novel example of “periosteal reaction”—new growth of the bone surface in response to various stimuli such as infection, injury, stress, or malignancy.^40^

In vitro, PSCs isolated from the mandible exhibit universal mesenchymal stem cell characteristics, as well as the expression of CTSK, a marker of the periosteal mesenchyme.^41^ ABL promotes proliferation, migration, and collagen synthesis in PSCs. Nevertheless, ABL alone fails to promote the subsequent ossification. Interestingly, the conditioned media from the osteocyte-like cells enhance mineralization of the PSCs, which is even promoted when combined with ABL. These imply an underlying role of osteocytes in assisting osteogenesis in the PSCs. Osteocytes are the most abundant cells in bone and are embedded within the mineralized bone matrix. It is well established that osteocytes respond to mechanical and hormonal stimuli and, in turn, secrete molecules that affect the effector cells by paracrine mechanisms.^42^ Specifically, PTH1R is recognized as an integrator of hormonal and mechanical signals converging in osteocytes.^8^ Therefore, the ABL-induced osteogenesis in alveolar bone may result from the orchestration of PSCs and osteocytes, though the specific mechanisms remain to be elucidated in the future.

This study revealed different roles of FAK in the ABL-induced osteogenesis in alveolar bone and limb bone. In the alveolar bone, FAKi completely blocked the ABL-induced osteogenesis, whereas it didn’t affect the ABL-induced osteoclastogenesis, suggesting that FAK may mediate only the anabolic but not catabolic role of ABL. In the limb bone, FAK also takes part in mediating the ABL-induced cortical bone thickening at specific parts, however, playing a less crucial role. Notably, the calvarial and limb bone osteocytes demonstrate differential expression of mechanoresponse-related genes, enriched for the FA pathway,^43^ implying their dissimilar roles in the bone development from intramembranous or endochondral ossification.

FAK is an essential component of the FA pathway and mediates diverse downstream signaling, including cell migration, proliferation, adhesion, and differentiation.^44–46^ In a mouse mandibular distraction osteogenesis model, gradual distraction exhibits direct intramembranous ossification, in which the mechanotransduction of skeletal stem cells is mediated via FAK.^36^ FAK has been shown to modulate early bone progenitor cell proliferation^46^ and mesenchymal stem cell migration.^47^

FAK activation in human mesenchymal stem cells is primarily mediated through phosphorylation at Y397 and Y925, with Y576, 577 contributing to a lesser extent, in osteogenic differentiation.^48^ In the mandibular distraction osteogenesis model, inhibition of Y397 phosphorylation has been shown to stunt skeletal lineage differentiation and progenitor expansion.^36^ In this study, we employed PF-573228, a selective inhibitor of FAK Y397 phosphorylation, and demonstrated that blockade of this site abolished the ABL-induced alveolar bone formation. Future studies may delve into other phosphorylation sites of FAK in the ABL scenario to gain a more comprehensive understanding of the underlying mechanisms.

The potential interplay between the PTH1R and FAK signaling pathways has been illuminated in tumor research. In human melanoma cells, PTHrP knockout or anti-PTHrP monoclonal antibody reduced both total FAK protein and phosphorylation at Y397. PTHrP (1–34) treatment partially rescued both the total and phosphorylated FAK levels, suggesting that PTHrP may regulate FAK expression and activation.^49^ In human colon cancer cell lines (Caco-2 and HCT116), exogenous PTHrP upregulated FAK via the ERK/RSK pathway, rather than the p38 MAPK pathway, thereby promoting cell migration.^50^

Additionally, PTH1R and FAK may share multiple signaling pathways, suggesting potential crosstalk between them. Activation of PTH1R may trigger downstream pathways such as Wnt/β-catenin,^51^ MAPK,^52^ and PI3K/Akt/mTOR,^53,54^ known to regulate cell proliferation, osteoblast differentiation, and migration. Notably, these pathways are also downstream effectors of FAK, a critical mechanotransduction component. Wnt/β-catenin and MAPK pathways are modulated by FAK to regulate proliferation, migration, and osteogenic differentiation in synovial mesenchymal stem cells.^55^ Under oscillatory shear stress, FAK activates the PI3K/Akt/mTOR pathway to promote osteoblast proliferation.^56^ Moreover, PTH1R signaling upregulates Src activity,^57^ which can activate FAK by phosphorylating its key tyrosine residues and form the FAK–Src complex to initiate downstream signaling.^44^

To summarize this study, intraoral submucosal injection of ABL, when combined with sufficient mechanical stimulation, promotes significant alveolar bone thickening via periosteal osteogenesis, which could be mediated by FAK activity. It presents a promising therapeutic approach for in situ alveolar bone augmentation by local drug administration, which may prevent or repair the detrimental periodontal dehiscence, potentially beneficial for orthodontic treatment.

## Materials and methods

### LM model and BM model in rats

Sprague-Dawley male rats (9 weeks, 200–250 g) were anesthetized by intraperitoneal injection of 50 mg/kg pentobarbital and fixed on the operating panel to install the LM or BM devices. The LM model was developed as previously described,^18^ using an inclined bite splint to apply labial force to the mandibular incisors (Fig. S1a). For the BM model, a 2 × 2 × 4 mm^3^ tube was bonded on the lingual surfaces of the maxillary molars, and a 50 cN buccal force was applied by inserting a double helix spring (Fig. S1c). All animal experiments were approved by the Research Ethics Committee of West China Hospital of Stomatology, Sichuan University, China (approved number: WCHSIRB-D-2022-402).

In the LM model, rats were randomly divided into the Ctrl, LM, ABL, and ABL + LM groups. Under isoflurane inhalation anesthesia, ABL (20 μg/kg) was injected daily submucosally at the labial vestibule of the mandibular incisors. In the BM model, ABL (20 μg/kg) was injected on the right side while saline was injected on the left side at the buccal vestibular sulcus between the maxillary first and second molars. After a 2-week intervention, all rats were euthanized at 11 weeks, and their maxillae (BM model), mandibles (LM model), and femurs (LM model) were harvested. For RNA sequencing, 12 rats were randomly divided into the Ctrl, LM, ABL, and ABL + LM groups (*n* = 3 in each). After treatment, they were euthanized, and labial alveolar bone was collected for further analysis.

Animals were monitored daily for general health, including body weight, food and water intake, and any signs of distress or discomfort. Special attention was paid to the oral cavity and the region surrounding the orthodontic device for signs of inflammation, infection, or detachment. Additionally, topical lidocaine (2%) was applied to the mucosal surface to reduce pain from the local injection.

### RNA-seq and analysis

Total RNA was extracted from the mandibular labial alveolar bone using TRIzol reagent kit (Invitrogen, USA). RNA integrity was assessed using the RNA Nano 6000 Assay Kit of the Bioanalyzer 2100 system. Then, a cDNA library was assessed on the Agilent Bioanalyzer 2100 system. The Illumina Novaseq platform was utilized for high-throughput RNA-seq. The DESeq2 R package was used to identify differentially expressed genes between samples from each group. Finally, the clusterProfiler package was used to perform the statistical enrichment of differential expression genes in GO and KEGG pathways.

### Micro-CT scanning

Scanning was performed with the μCT50 instrument (Scanco, Switzerland) at a 15 μm resolution for quantitative analysis.

For the mandibular labial alveolar bone, the mid-ridge level was set at the attachment point of the digastric muscle, while the crest level was at the anterior quarter of the alveolar crest. Alveolar bone thickness perpendicular to the tooth root surface was measured at both levels, and an area of 250 × 250 μm^2^ in 50 slices (750 μm) was selected as the region of interest (ROI) for BMD measurement (Fig. S1b). For the maxillae, the distobuccal root of the first maxillary molar was the anatomical landmark, and alveolar bone thickness was measured at 100 μm below the alveolar ridge crest (crest level) and at the height of the mid-root (mid-ridge level), with an area of 330 × 330 μm^2^ in 50 slices (750 μm) selected as the ROI (Fig. S1d). For the femur, trabecular bone was analyzed in the distal femoral metaphysis, and cortical bone in the femoral diaphysis and distal metaphysis (Fig. S1e), following established micro-CT guidelines for rodent bone.^58^

### Histological analysis

After micro-CT scanning, samples were decalcified in 10% EDTA for 6 weeks. Serial sections (5-μm-thick) were performed. For H&E staining, sections were deparaffinized, rehydrated, and stained with hematoxylin (5 min) and eosin (2 min). For Masson staining, sections were stained using a commercial kit (Solarbio, China) following the manufacturer’s protocol. Sections were also used for TRAP staining according to the manufacturer’s instructions (Sigma–Aldrich, USA). Images were captured using a Leica DM 2500 microscope. The bone tissue width and TRAP (+) cell number were measured using ImageJ software.

### Immunohistochemical assay

Sections were incubated overnight with primary antibodies against p-FAK (1:100, Huabio, ET1610-34), FAK (ET1602-25, 1:50, Huabio), COL1A1 (ET1609-68, 1:200, Huabio), and OCN (ER1919-20, 1:200, Huabio), followed by horseradish peroxidase (HRP)-coupled secondary antibodies. HRP activity was detected using 3,3′-diaminobenzidine (DAB) for 3–8 min followed by hematoxylin counterstaining. Images were acquired using a Leica DM 2500 microscope. Quantification was performed in the ImageJ software using the “Color Deconvolution” plugin with the “H DAB” vector to isolate the DAB signal from hematoxylin. Integrated optical density (IOD) and positive staining area were measured, and the mean optical density (MOD) was calculated as: MOD = IOD/area. Each sample was represented by the average MOD value calculated from three randomly selected, non-overlapping fields of view.

### PSC culture and characterization

PSCs were isolated by a whole-bone periosteal digest.^59,60^ Mandibles from 2-week-old male rats were dissected free of muscle and connective tissue before being subjected to enzymatic digestion with serum-free α-MEM medium containing Collagenase (1 mg/mL), Dispase II (2 mg/mL), and DNase I (1 mg/mL) at 37 °C for 30 min. To prevent contamination by residual muscle and connective tissue cells, cells obtained from the first two digestions were discarded. After a subsequent 1-h digestion, the digest was centrifuged and resuspended in growth medium (α-MEM containing 1% penicillin/streptomycin [PS] and 10% fetal bovine serum [FBS]). Osteogenic medium consisted of 5% FBS, 1% PS, 10 mmol/L β-glycerophosphate, 50 μg/mL ascorbic acid, and 100 nM dexamethasone, was used for osteogenic induction of PSCs. Adipogenic and chondrogenic differentiation followed commercial kit protocols (RAXMX-90031 and RAXMX-90041, Cyagen, China).

### Flow cytometric analysis

Cells were incubated with fluorescein isothiocyanate (FITC)-conjugated monoclonal antibodies specific to CD29 (E-AB-F1309E, Elabscience), CD44 (E-AB-F1225C, Elabscience), CD45 (E-AB-F1227C, Elabscience), and CD90 (E-AB-F1226C, Elabscience) in Flow Cytometry Staining Buffer (00-4222-57, Thermo Fisher) for 30 min at 4 °C. Cells were fixed and permeabilized with IC Fixation Buffer (FB001, Thermo Fisher) and Permeabilization Buffer (00-8333-56, Invitrogen), followed by staining with anti-CTSK antibody (ABCCS27193, ABCbiolab) in 1× Permeabilization Buffer for 1 h. Samples were analyzed using the Beckman CytoFLEX Flow Cytometer and processed with FlowJo software.

### MLO-Y4 cell culture and CM collection

Osteocyte-like MLO-Y4 cells were plated in collagen-coated 6-well plates at a density of 4 × 10^5^ cells/well. After 48 h of culture, the cells were washed once with warm PBS before the medium was changed into serum-free α-MEM for an additional 24 h incubation, following which the CM was collected and stored at −80 °C until subsequent use.

### ABL and CM administration in vitro

Intermittent ABL administration was employed to play its anabolic role.^61^ PSCs were exposed to ABL (100 ng/mL) for the first 6 h in each 48-h incubation cycle, after which the medium was replaced with fresh osteogenic medium without ABL for the remaining 42 h. In the co-stimulation experiment, the induction of osteogenic differentiation was performed using the mixture of 50% CM and 50% fresh osteogenic medium^62^ with or without ABL. ABL was also only used for the first six hours.

### FAK inhibitor treatment in vivo and in vitro

FAK inhibitor PF-573228 (A230913, Ambeed, USA), targeting FAK phosphorylation at Tyr397, was utilized both in vivo and in vitro. Based on a previous study,^36^ 50 μmol/L PF-573228 was selected for its efficacy and low toxicity. In vivo, after establishing the BM models, 10 rats were injected with PF-573228 at 50 μmol/L in a 100 μL volume into the right buccal submucosa, with DMSO injected on the left side. After 6 h, 5 rats were injected with ABL (20 μg/kg) and 5 with saline into the submucosa bilaterally. Additionally, PF-573228 was injected intraperitoneally, followed by a subcutaneous injection of ABL (80 μg/kg) 6 h later. Injections were administered twice daily for 2 weeks before tissue harvest. In vitro, 50 μmol/L PF-573228 was added at each medium change over the 48 h cycle.

### CCK-8

CCK-8 is a colorimetric assay used to evaluate cell viability and proliferation based on dehydrogenase activity in living cells. PSCs were seeded in 96-well plates (5 × 10^3^ cells per well) and received different drug treatments for 1, 2, and 4 d. Then, CCK-8 (10 µL, APExBIO, USA) was added to each well and incubated for 1 h at 37 °C. Optical density (OD) value at 450 nm was measured by a microplate reader.

### CFU assay

PSCs were seeded in 24-well plates (250 cells per well) and then treated with corresponding drugs for 4 d. Subsequently, the cells were washed with PBS, fixed with 4% PFA for 20 min, and stained with crystal violet solution.

### Wound healing assay

PSCs were seeded in 6-well plates (1 × 10^6^ cells per well), and scratches were made perpendicular to the long axis. Cells were treated with different drugs in serum-free α-MEM. Cell migration was photographed at 0 h and 24 h after scratching using a microscope, and scratch areas were measured using ImageJ. Wound closure (%) was calculated as: (area at 0 h – area at 24 h) / area at 0 h.

### ALP staining and ARS staining

After 6 days (three 48 h cycles), PSCs were fixed with 4% PFA for 20 min and stained for ALP using a BCIP/NBT ALP color development kit (Beyotime, China). After 14 days (seven cycles), extracellular matrix calcification was assessed with 1% Alizarin Red S (Solarbio, China) for 15 min.

### RNA isolation and real-time PCR analysis

Total RNA was isolated from PSCs using RNA-Quick purification kit (ES Science, China) and reverse-transcribed into cDNA with the PrimeScript RT Reagent kit (TaKaRa, Japan). Real-time PCR was performed using the TB Green qPCR kit (TaKaRa, Japan) on the StepOne Real-Time PCR System (Applied Biosystems, USA). GAPDH was used as an internal control gene, and relative gene expression was calculated using the 2^−ΔΔCt^ method. Primer sequences are listed in Table S1.

### Western blotting

Cells were lysed using RIPA buffer supplemented with protease and phosphatase inhibitor cocktails (1:100, APExBIO, USA) on ice. Lysates were centrifuged, and the supernatants were quantified by BCA assay (Beyotime, China). Protein was separated using an 8%–10% SDS gel and transferred onto a PVDF membrane. Membranes were then incubated overnight at 4 °C with primary antibody and incubated with HRP-conjugated secondary antibody for 1 h at room temperature. Primary antibodies were anti-p-FAK (ET1610-34, 1:1 000), anti-FAK (ET1602-25, 1:1 000), anti-COL1A1 (ET1609-68, 1:1 000), anti-RUNX2 (ET1612-47,1:2 000), and anti-GAPDH as a control (ET1601-4, 1:10 000), all from Huabio. Chemiluminescent signals were detected using the Bio-Rad Imaging System and quantified with ImageJ.

### Statistical analysis

Data are presented as mean ± standard deviation (SD). Statistical analysis was performed using GraphPad Prism 9. For normally distributed data, unpaired Student’s t-test (2 groups) or analysis of variance (ANOVA) with Tukey’s multiple-comparison test (≥3 groups) was used. For non-normally distributed data, the Kruskal–Wallis test was used. P-value less than 0.05 was considered statistically significant: *P < 0.05, **P < 0.01, ***P < 0.001.

## Supplementary information

Supplemental Material

## Data Availability

The data presented in this study are available upon request from the corresponding author.
