# Supplementary material for: Local abaloparatide administration promotes in situ alveolar bone augmentation via FAK-mediated periosteal osteogenesis
Source: Int J Oral Sci. 2025 Sep 2;17:63. doi: 10.1038/s41368-025-00392-6 (PMC12402510; doi:10.1038/s41368-025-00392-6)
Supplement: Supplementary file 1 — Supplemental Material [file 41368_2025_392_MOESM1_ESM.docx]

Supplementary Figures


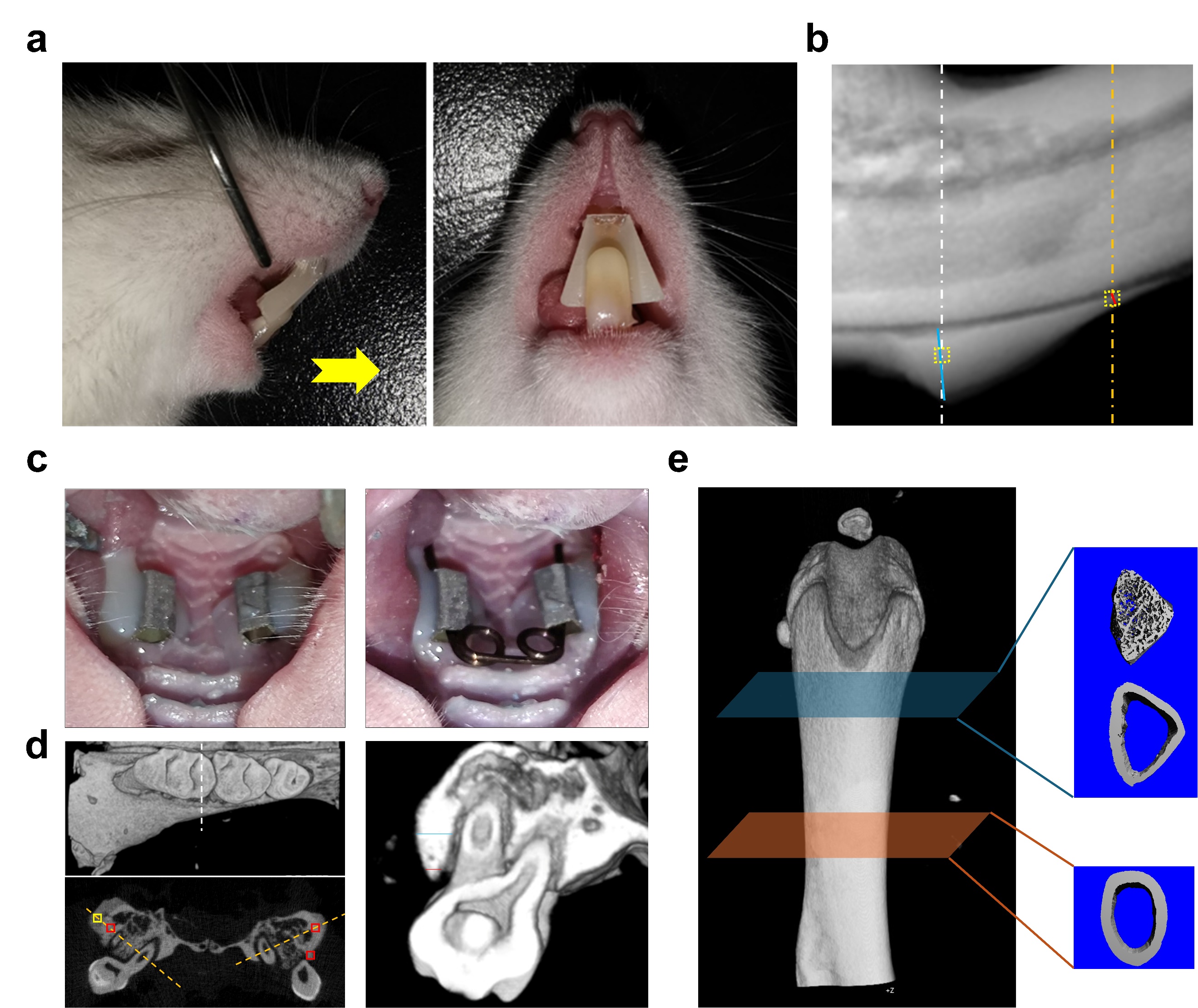


**Figure S1 Models and analysis for labial alveolar bone, buccal alveolar bone, and femur.** **a** LM model: created with a bite splint applying labial force (yellow arrow) on mandibular incisors. **b** Micro-CT of labial alveolar bone: orange/white dashed line: crest/mid-ridge level; red/blue solid lines: bone thickness at the crest/mid-ridge level; yellow box: ROI for BMD analysis. **c** BM model: established with a maxillary expansion device. **d** Micro-CT of buccal alveolar bone: white dashed line: plane of distobuccal root of the maxillary first molar; orange dashed line: the mid-ridge level; red/blue solid line: bone thickness at the crest/mid-ridge level; red box: ROI for BMD at the crest and mid-ridge levels; yellow box: ROI for BMD of the newly formed bone. **e** ROI for micro-CT analysis of femur. Blue/orange cut plane: the femoral distal metaphyseal/diaphyseal region for trabecular and cortical analysis.


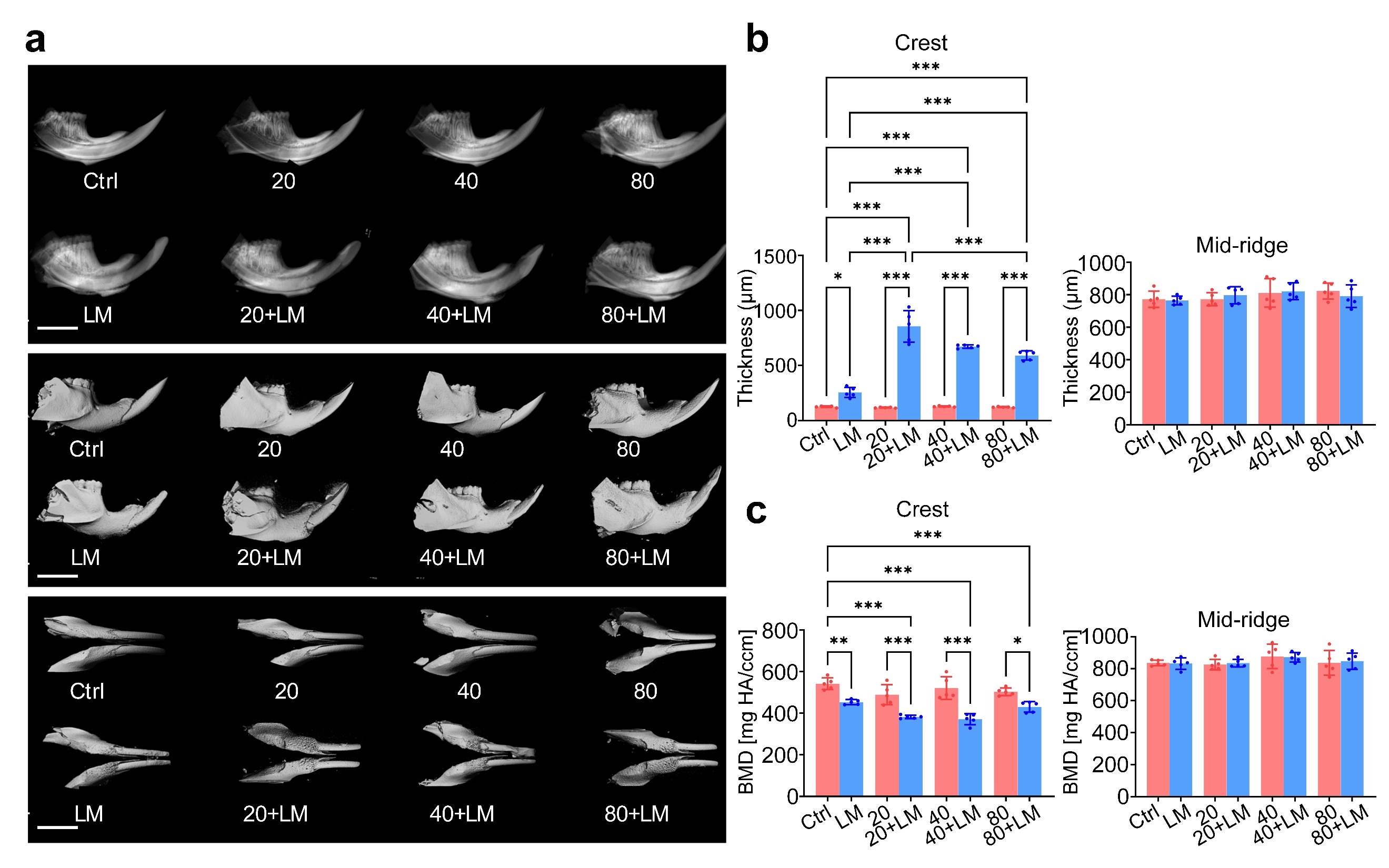


**Figure S2. Effects of local ABL injection at different dosages in the LM model.** **a** Micro-CT reconstructed images. Scale bar: 5 mm. **b** Alveolar bone thickness at the crest and mid-ridge levels. **c** BMD of the alveolar bone at the crest and mid-ridge levels. ABL dosages: 20 (20 μg/kg), 40 (40 μg/kg) and 80 (80 μg/kg). *p < 0.05, **p < 0.01, ***p < 0.001.


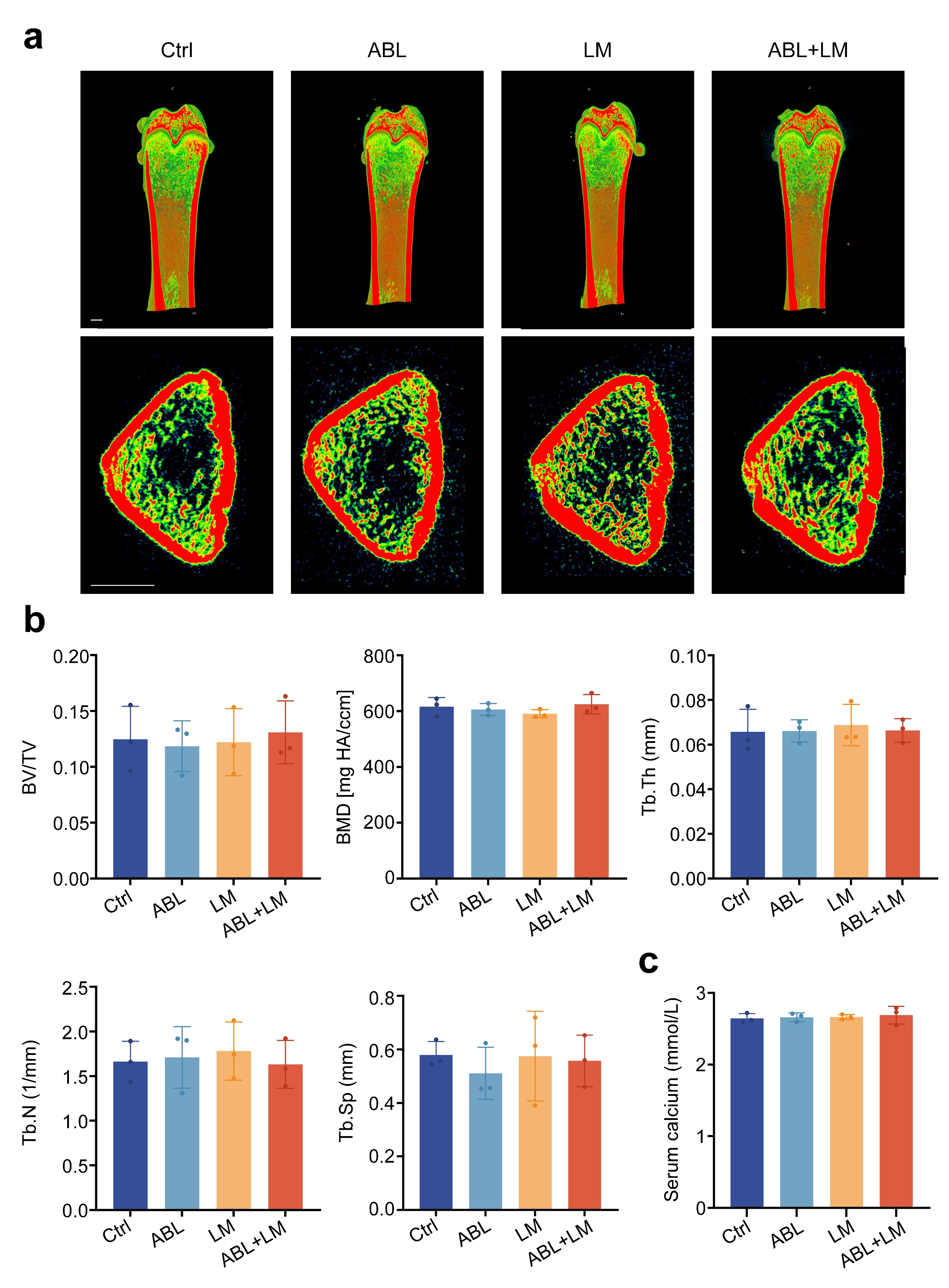


**Figure S3. Systemic safety of locally injected ABL (20 μg/kg) confirmed by evaluation of femur density and serum calcium in the LM model.** **a** Micro-CT reconstructed images of femur. Scale bar: 1mm. **b** Trabecular analysis of femur. **c** Serum calcium.

**Figure S4. Immunohistochemical staining and quantitative analysis of mandibular labial alveolar bone. a** COL1A1. **b** Ki-67. **c** OCN. Scale bar: 100 μm. B: alveolar bone; P: periosteum. *p < 0.05, **p < 0.01, ***p < 0.001.


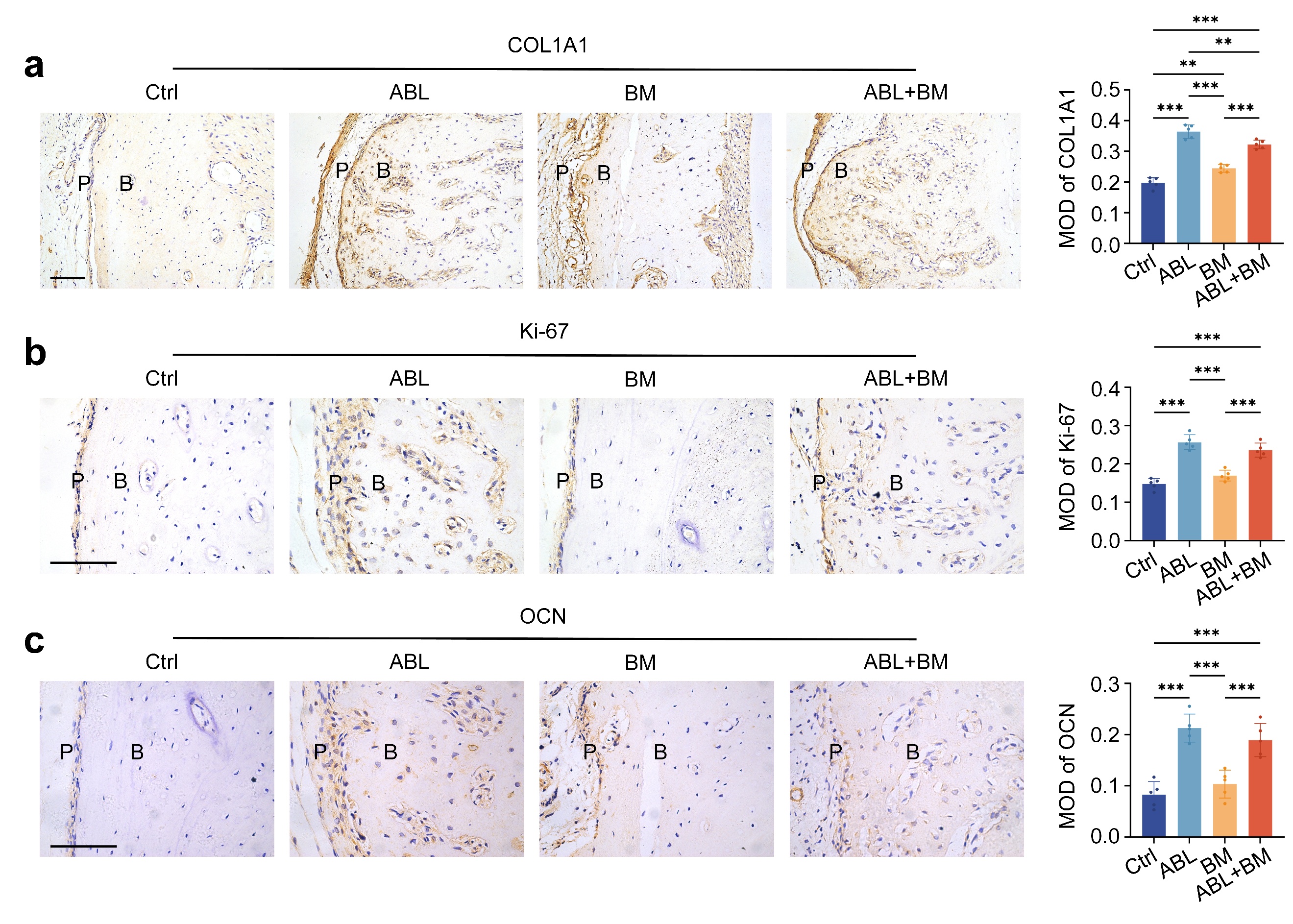


**Figure S5. Immunohistochemical staining and quantitative analysis of maxillary buccal alveolar bone. a** COL1A1. **b** Ki-67. **c** OCN. Scale bar: 100 μm. B: alveolar bone; P: periosteum. *p < 0.05, **p < 0.01, ***p < 0.001.


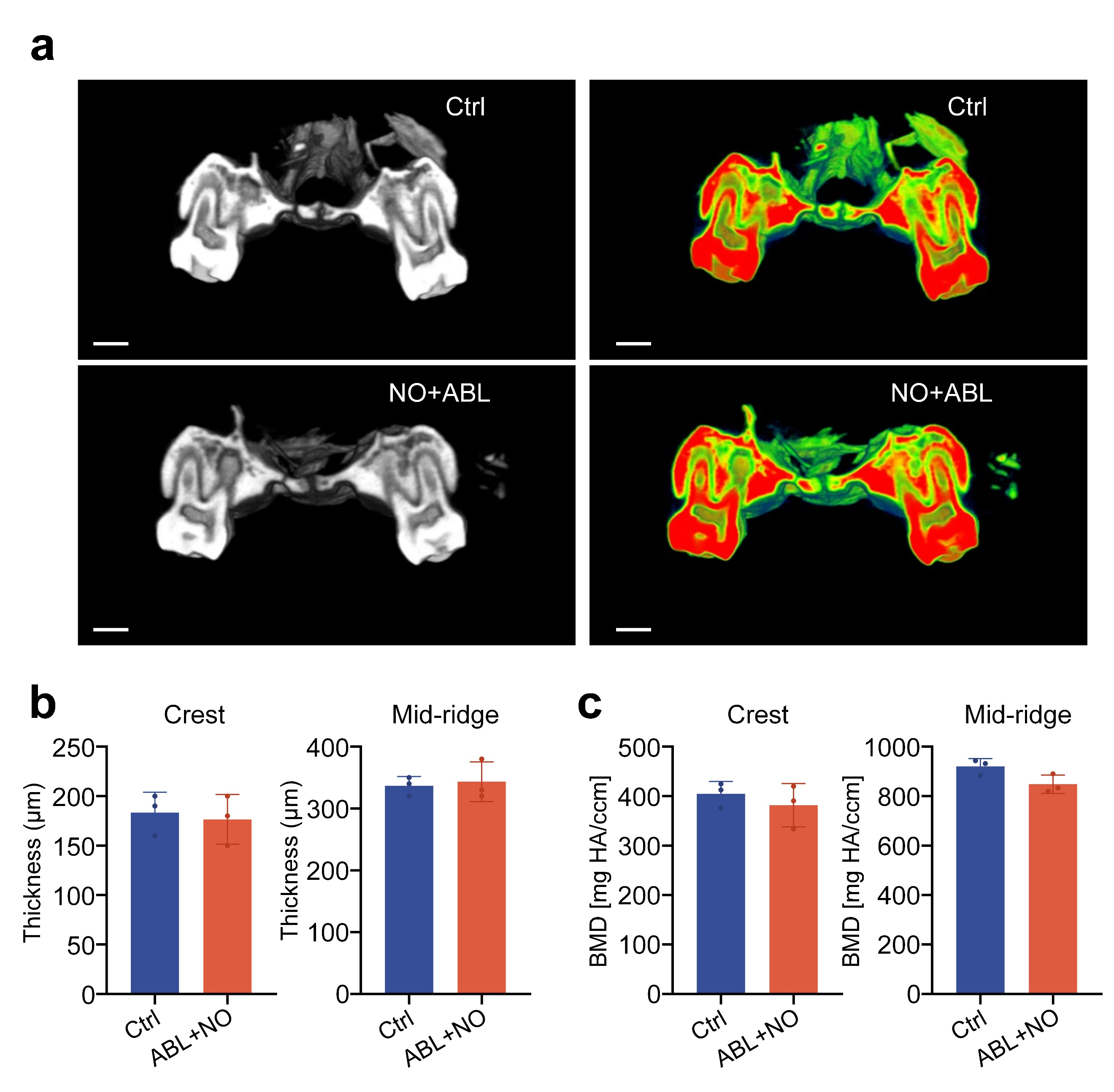


**Figure S6. The effects of local ABL injection on the buccal alveolar bone in the non-occlusion (NO) model.** NO model was constructed by extracting the right mandibular molars, followed by ABL injection at the right maxillary buccal vestibule. **a** Micro-CT reconstructed images. Scale bar: 1mm. Alveolar bone thickness (**b**) and BMD (**c**) at the crest and mid-ridge levels.


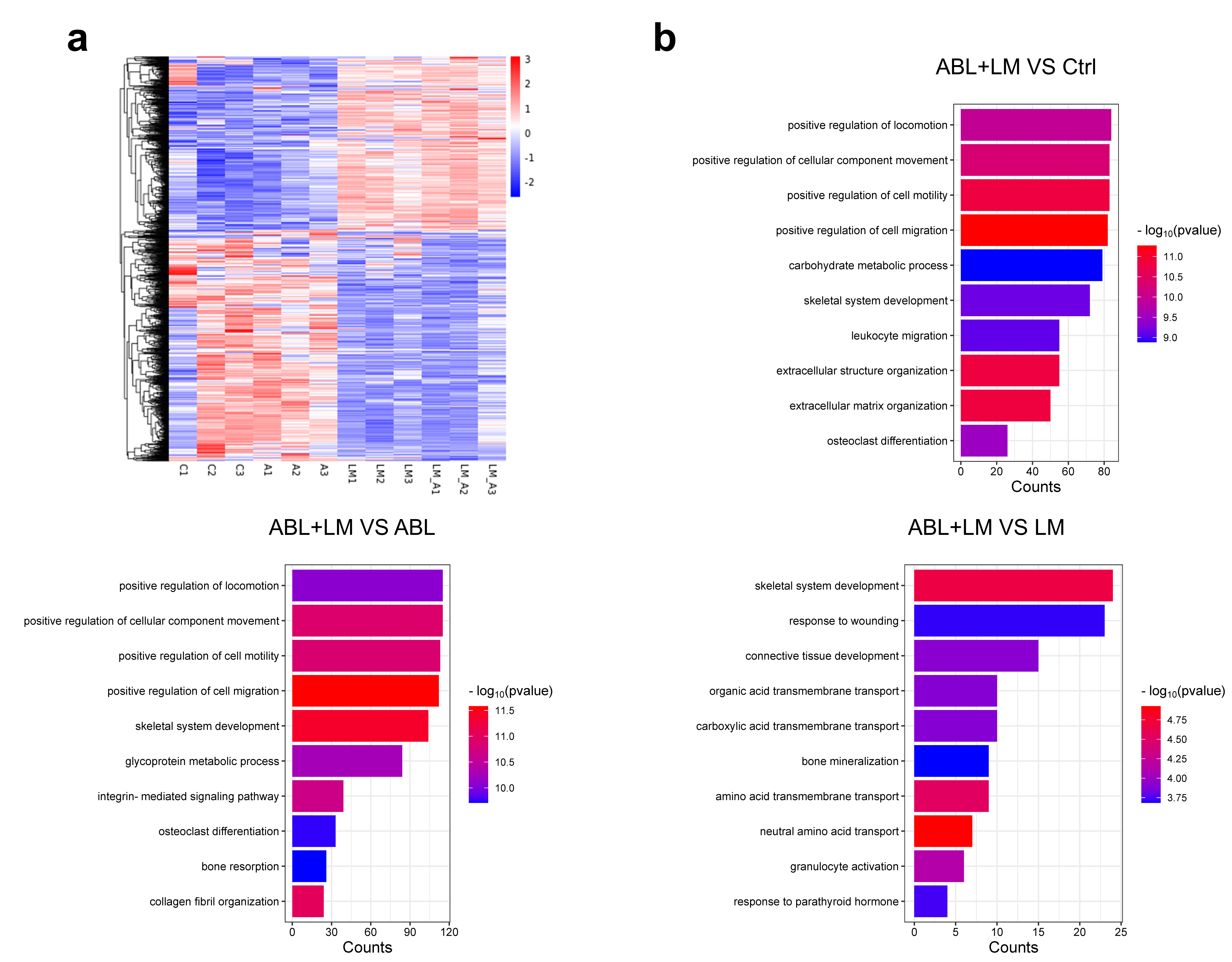


**Figure S7. Differential gene expression and GO analysis of mandibular labial alveolar bone in LM model by RNA-Seq. a** Heatmap. **b** GO analysis. The top ten upregulated GO terms in the ABL+LM group compared to other groups.


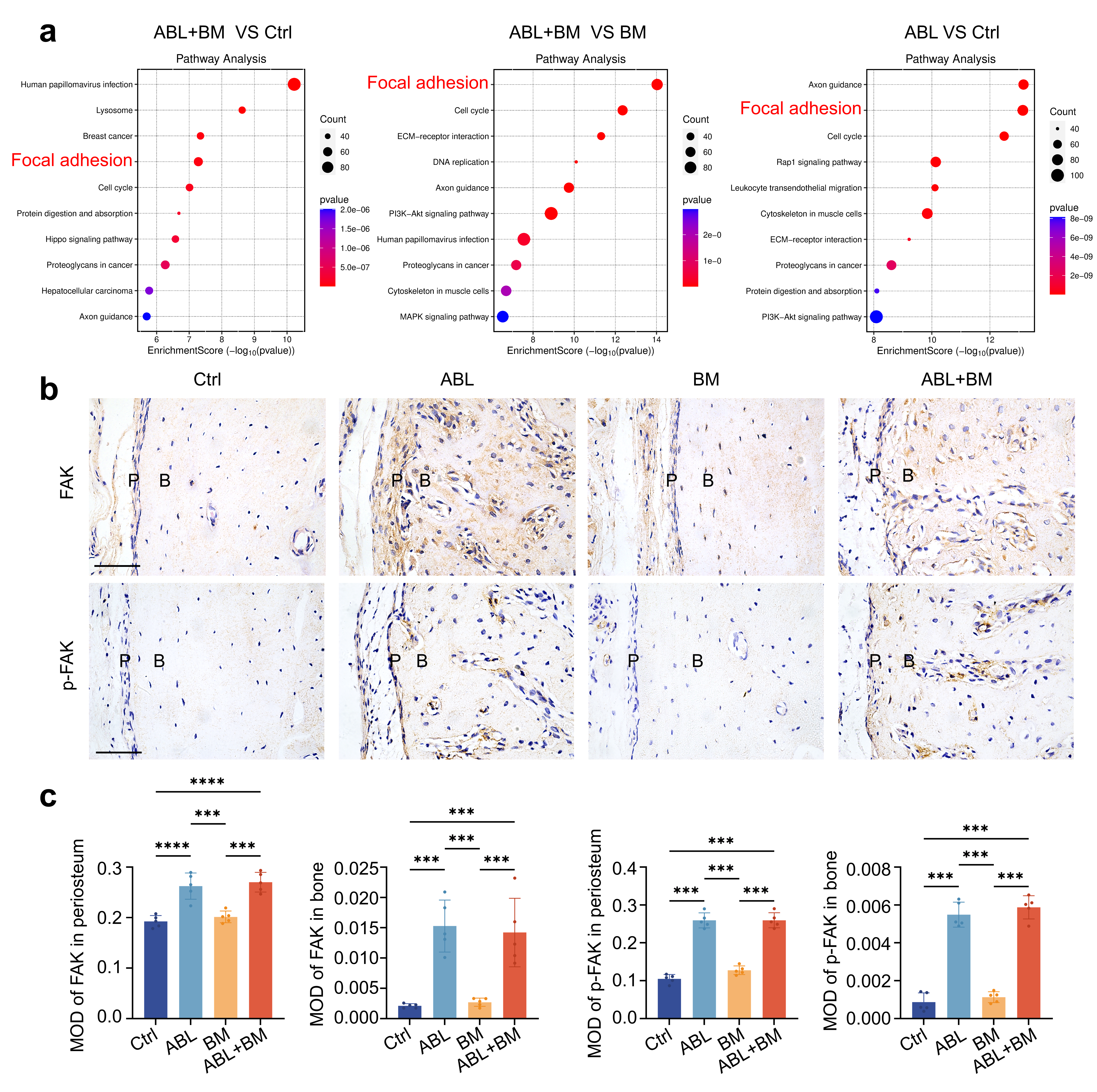


**Figure S8. KEGG pathway analysis of maxillary buccal alveolar bone in BM model and validation by IHC. a** KEGG pathway enrichment analysis in the BM model. Focal adhesion is among the top five pathways enriched in the ABL+BM vs. Ctrl, ABL+BM vs. BM and ABL vs. Ctrl comparisons. **b** IHC staining of FAK and p-FAK in maxillary buccal alveolar bone, with quantification in (**c**). B: alveolar bone; P: periosteum. *p < 0.05, **p < 0.01, ***p < 0.001.


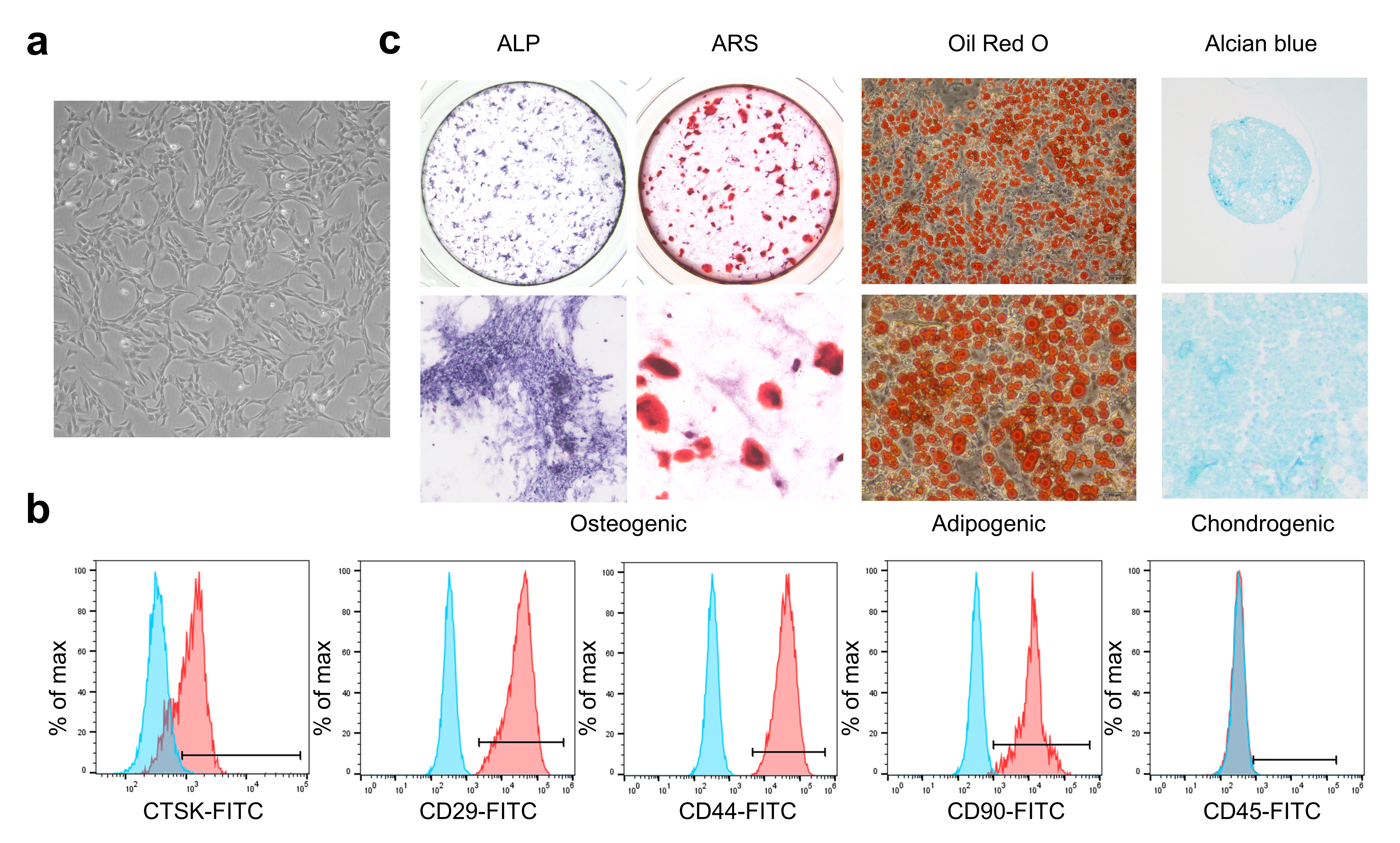


**Figure S9. Characterization of PSCs.** **a** Morphology of PSCs **b** Flow cytometric analysis. **c** tri-lineage differentiation of PSCs.


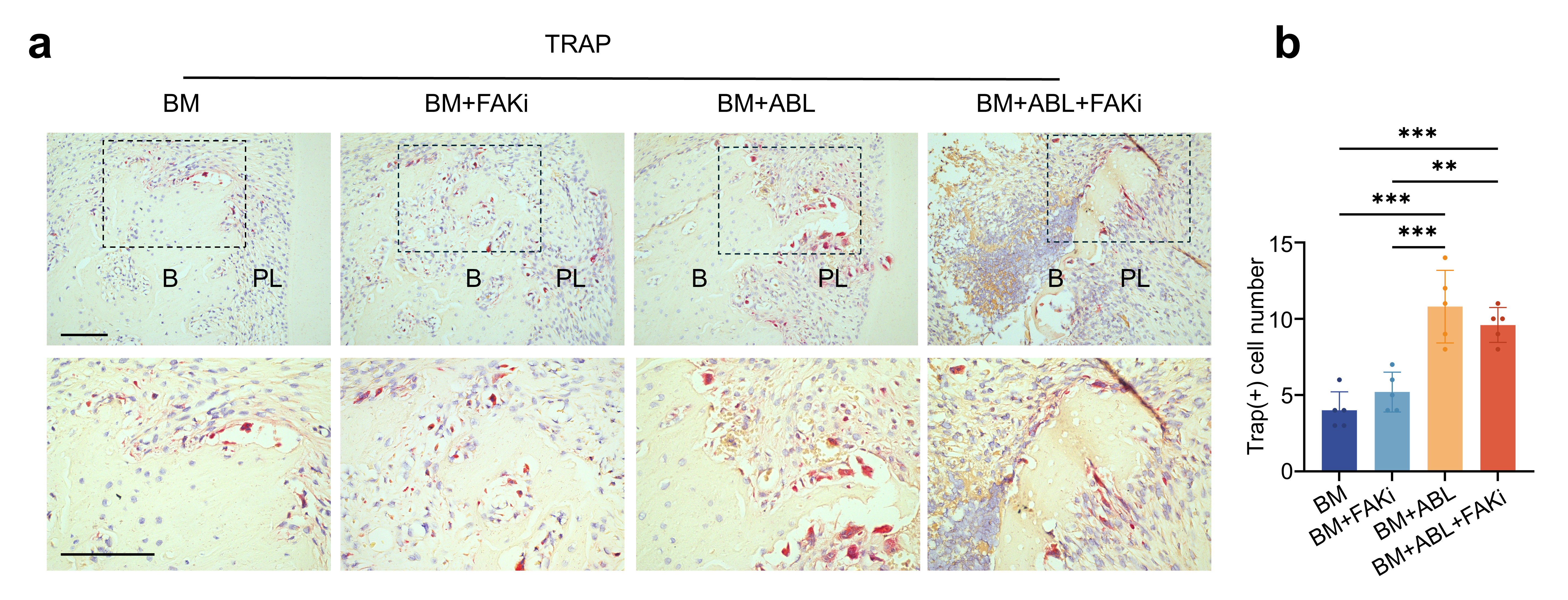


**Figure S10. Inhibition of FAK activity dose not affect the catabolic effect of ABL a** TRAP staining images. Scale bar: 100 μm. **b** Quantitative analysis of TRAP+ cells on the periodontal side of buccal alveolar at 2 weeks after drug injection. PL: periodontal ligament; B: alveolar bone. n=5 per group. *p < 0.05, **p < 0.01, ***p < 0.001.

**Supplementary Table**

**Table S1. Primer sequences of RT-qPCR**

| Target gene | Forward primer (F) 5′-3′ | Reversed primer (R) 5′-3′ |
| --- | --- | --- |
| *Alp* | CACGGCGTCCATGAGCAGAAC | CAGGCACAGTGGTCAAGGTTGG |
| *Col1a1* | TGTTGGTCCTGCTGGCAAGAATG | GTCACCTTGTTCGCCTGTCTCAC |
| *Gapdh* | AAGATGGTGAAGGTCGGTGT | GCTTCCCATTCTCAGCCTTG |
| *Runx2* | CTCTTCCCAAAGCCAGAGCG | ACCATCCTGGAAGGAGACCG |
| *Fak* | CACCTGATGGAAGAGCGGCTAATC | GGATCGGTCAAGGTTGGCAGTG |
